# Near-wins and near-losses in gambling: A behavioral and facial EMG study

**DOI:** 10.1111/psyp.12336

**Published:** 2014-09-19

**Authors:** Yin Wu, Eric van Dijk, Luke Clark

**Affiliations:** a Behavioural and Clinical Neuroscience Institute, Department of Psychology, University of CambridgeCambridge, UK; b Department of Social and Organizational Psychology and Leiden Institute for Brain and Cognition, Leiden UniversityLeiden, The Netherlands; c Centre for Gambling Research at UBC, Department of Psychology, University of British ColumbiaVancouver, British Columbia, Canada

**Keywords:** Electromyography, Risk taking, Cognitive distortion, Near-miss, Gambling

## Abstract

This study investigated responses to near-wins (i.e., nonwin outcomes that were close to a major win, and their counterpart, near-losses (nonwin outcomes that are proximal to a major loss) in a decision-making task, measuring (a) luck ratings, (b) adjustment of bet amount, and (c) facial muscle reactivity at zygomaticus and corrugator sites. Compared to full-misses, near-wins decreased self-perceived luck and near-losses increased self-perceived luck, consistent with the effects of upward versus downward counterfactual thinking, respectively. Wins and losses both increased zygomaticus reactivity, and losses selectively enhanced corrugator reactivity. Near-wins heightened zygomaticus activity, but did not affect corrugator activity, thus showing a similar response pattern to actual wins. There were no significant facial EMG effects of near-losses. We infer that near-wins engender some appetitive processing, despite their objective nonwin status.

Gambling is a widespread form of entertainment where a monetary wager is placed upon the uncertain prospect of a larger monetary win. Its allure can provide insight into the psychological mechanisms of human decision making. Previous research has shown that near-wins—nonwin outcomes that are proximal to a jackpot—foster persistent play (Côté, Caron, Aubert, Desrochers, & Ladouceur, 2003, Kassinove & Schare, 2001) and increase motivational ratings (Clark, Lawrence, Astley-Jones, & Gray, 2009). Slot machine near-wins were perceived as being “closer” to wins than to losses (Dymond et al., 2014). Using functional magnetic resonance imaging, near-wins were also found to increase neural signal in brain reward circuitry that overlapped with the jackpot wins (Chase & Clark, 2010; Clark et al., 2009). Nevertheless, near-wins also have a negative emotional component; for example, they are rated as significantly less pleasant than full-miss outcomes (Clark, 2010; Clark et al., 2009, 2013; Qi, Ding, Song, & Yang, 2011).

Psychophysiology may provide a useful tool for further characterizing the bivalent emotional response to these events. Past work has shown that near-wins increase electrodermal activity (EDA) and heart rate acceleration, in comparison to full-miss outcomes (Clark, Crooks, Clarke, Aitken, & Dunn, 2012; Clark et al., 2013; Dixon et al., 2011). EDA is a sensitive marker of physiological arousal that responds to both aversive and appetitive stimuli, and thus offers limited valence specificity (Dawson, Schell, & Filion, 2007; Lobbestael, Arntz, & Wiers, 2008). Phasic heart rate changes do show some differences by valence (e.g., Bradley, Codispoti, Cuthbert, & Lang, 2001), but this is a complex multiphasic response that also varies across individuals (Hodes, Cook, & Lang, 1985). Facial muscle activity offers an alternative probe of stimulus-evoked emotional reactivity with superior valence differentiation, with zygomaticus activity (recorded on the cheek) linked to appetitive processing, and corrugator supercilii activity (recorded on the eyebrow) scaling with aversive processing (Cacioppo, Petty, Losch, & Kim, 1986; Lang, Greenwald, Bradley, & Hamm, 1993). The present study employed facial electromyography (EMG) at these two sites to better decompose the bivalent emotional nature of gambling “near” events.

Little attention has been paid to the natural counterpart to the near-win, the “near-loss.” In a decision with the possibility of losing money, what happens if you discover that you narrowly missed a major loss? While these events are less ubiquitous in gambling behavior, they do occur across many areas of day-to-day decision making, for example, when we narrowly miss an accident, or traffic jam, and they have received some attention in occupational psychology. For example, narrowly avoiding a great disaster lowered the future perceived risk of that event occurring, and increased risky choice (Dillon & Tinsley, 2008). The present study sought to model these events in a gambling situation, by developing a wheel of fortune task in order to deliver both near-wins versus near-losses in the same environment. Our design was based upon a “single-shot” game by Wohl and Enzle (2003) in which participants experiencing a near-loss were more likely to bet on a second risky prospect. In order to quantify psychophysiological reactivity to the gambling outcomes (as well as behavioral measures), we developed a multishot version of their procedure.

The impact of these near events may be understood from the perspective of counterfactual thinking, the mental processes by which people consider salient alternatives to events that actually occurred (Epstude & Roese, 2008; Roese, 1997; Zhang & Covey, 2014). Counterfactual thinking is seen to amplify emotional responses, and impacts upon behavioral regulation (see Epstude & Roese, 2008, for a review). In considering the counterfactual thoughts associated with near events, it is important to distinguish two types: upward counterfactuals involve unobtained outcomes that are better than what actually happened, whereas downward counterfactuals involve unobtained outcomes that are worse than reality (Roese, 1997). These directions may have distinct effects on emotional responses. While upward counterfactual thinking is associated with a state of regret and negative affect, downward counterfactuals tend to elicit relief and positive affect (Roese, 1994).

As well as the counterfactual direction, outcome closeness is a further determinant of counterfactually driven emotions (Meyers-Levy & Maheswaran, 1992). In a scenario from Kahneman and Tversky (1982), participants indicated that Traveler A, who missed his flight by 5 min, would feel more upset than Traveler B, who missed his flight by 30 min. Thus, emotional reactions to a negative outcome may be intensified if the distance between the unobtained and obtained outcomes is close. To quantify these putative counterfactual thoughts to near-wins and near-losses in our gambling task, we administered trial-by-trial luck ratings. Perceptions of luckiness were shown previously to be sensitive to close counterfactuals (Teigen, 1995, 1996). We hypothesized that, compared to full-misses, near-losses would activate downward counterfactuals, and make individuals feel luckier. On the other hand, we expected that near-wins would elicit upward counterfactuals, and make people feel unlucky. To check our manipulation and to corroborate the counterfactual nature of these effects, we administered a questionnaire after participants completed the task, where we used screenshots of the different outcomes to ask about their first thoughts as to how the outcome could have been different (see also Wohl & Enzle, 2003).

As a second metric reflecting behavioral choice, participants also selected a bet on each trial. This enabled us to investigate the effects of near-wins and near-losses on subsequent gambling behavior. Darke and Freedman (1997a) found that the experience of a lucky event could make individuals feel more confident and bet more on a subsequent gamble, and that these effects were further moderated by the trait level of belief in luck. Priming participants with luck-related concepts also enhanced perceived luckiness and increased risky decision making (Jiang, Cho, & Adaval, 2009). In the present study, we reasoned that if near-wins and near-losses could influence self-perceived luck via counterfactual thinking, then this may modify risk-taking behavior on the subsequent trial. Specifically, we hypothesized that increased luck perceptions following near-losses would make individuals bet more in the following round, whereas decreased luckiness after a near-win would make individuals bet less. However, as previous research has shown that near-misses (i.e., near-wins) increased self-reported motivation to play (Clark et al., 2009, 2013), it is also possible that the appetitive aspect of near-wins could be manifested in an increased bet in the subsequent gamble.

Our predictions for the facial muscle reactivity were somewhat exploratory, given that only one prior study to our knowledge has examined facial EMG activity to gambling outcomes. Bediou, Mohri, Lack, and Sander (2011) found that, in the context of a competition task involving third-party arbitration decisions, large wins were associated with increased zygomaticus activity compared to large losses. Past work with a range of emotional stimuli shows corrugator responsivity to negative affect (Cacioppo et al., 1986; Lang et al., 1993), which we expected to generalize to financial losses. As such, we had a strong a priori hypothesis for the objective win and loss outcomes that wins would enhance zygomaticus activity, whereas losses would enhance corrugator activity. As emotionally complex events, we reasoned that near-wins would elicit aversive processing that would increase corrugator reactivity, and/or motivational processing that would increase zygomaticus activity. For near-losses, we predicted that the positive emotions associated with self-perceived luckiness would heighten zygomaticus activity as well.

## Method

### Participants

We recruited 45 healthy volunteers (25 men; mean age = 24.5, *SD* = 3.2) from the student population at the University of Cambridge for a study of gambling behavior. We determined this sample size based on previous facial EMG studies (Carr, Winkielman, & Oveis, 2013; Larsen, Norris, & Cacioppo, 2003). Our recruitment strategy excluded psychology and economics students, and was directed towards students with some interest in gambling by using an advertisement that asked, “Do you enjoy gambling?” At the end of the test session, participants completed three self-report instruments: (1) the gambling related cognitions scale (GRCS; Raylu & Oei, 2004) as an index of the trait susceptibility to gambling cognitions, and this scale indicated moderate levels of gambling involvement (*M* = 44.0, *SD* = 14.3, range 23–81) in the range of previous studies in recreational gamblers (Billieux, Van der Linden, Khazaal, Zullino, & Clark, 2012; Raylu & Oei, 2004); (2) the problem gambling severity index (Ferris & Wynne, 2001) to screen for problem gambling; no participants met the threshold for problem gambling (score ≥ 8; *M* = 0.53, *SD* = 0.94); (3) the belief in good luck scale (BIGL; Darke & Freedman, 1997b) to measure trait beliefs in luck. The study was conducted in accordance with the Declaration of Helsinki and was approved by the University of Cambridge Psychology Research Ethics Committee. Written informed consent was obtained from all participants. Volunteers attended individual testing sessions of 2-h duration, where they completed a computerized wheel of fortune task, with concurrent recording of facial EMG. All participants were paid the maximum possible win of £25 (approximately $37.80) as their reimbursement.

### Wheel of Fortune Task

**Figure 1:**
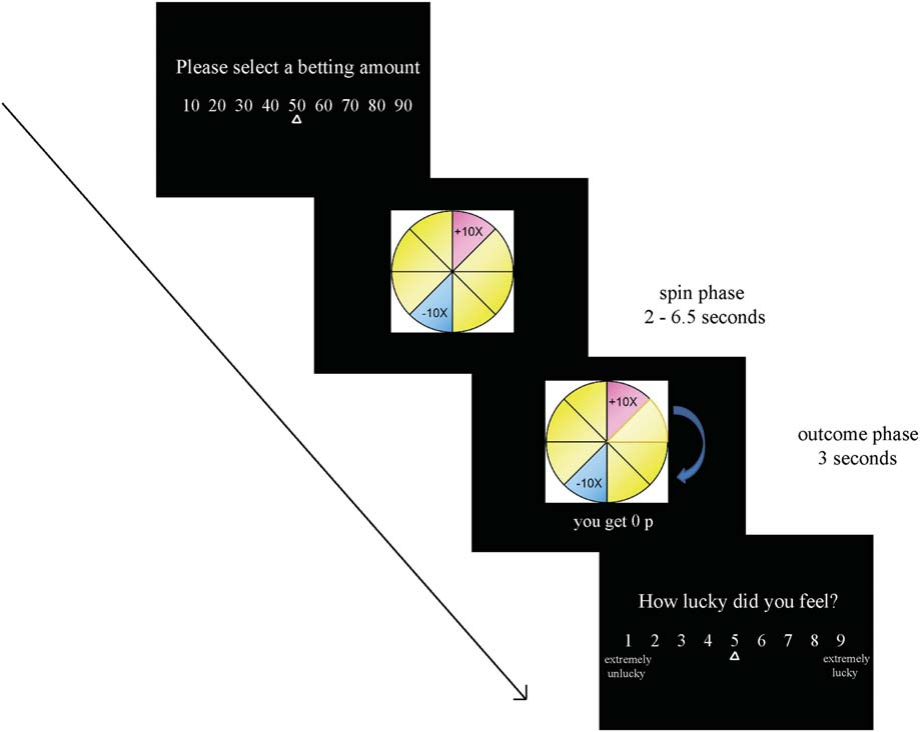
Sequence of events in a single trial. The arrow on the outcome phase indicates the direction of movement. This trial displays a near-win that has passed through the payline.

Participants completed 120 experimental trials on a computerized wheel of fortune task modified from Wohl and Enzle (2003; see Figure 1), and programmed using Presentation software (Neurobehavioral System Inc.). On each trial, the wheel was divided into eight segments of different colors. The + or − symbols in each segment indicated the amounts the participant stood to win or lose. Segments without any symbols represented zero outcomes (neither win nor lose). The number (e.g., 10) indicated the size of win/loss, as a multiplier of the amount that the participant bet^1^ on each round. For instance, +10× meant that the participant would win 10 times the wager, and −10× meant that he/she would lose 10 times the wager.

The specific trial timings were as follows (see Figure 1). At the beginning of each trial, the participant was asked to choose a bet between £0.10 and £0.90, in £0.10 increments. Following bet selection, the wheel spun for an anticipation interval (2–6.5 s), during which time the wheel decelerated to a standstill. The outcome phase then lasted 3 s, where the segment was highlighted, and there was accompanying auditory feedback (applause for winning outcomes, booing for losing outcomes, or neutral sounds for null outcomes), and the numeric outcome was displayed for 1 s. Following the outcome phase, a luck rating was displayed using a 9-point visual analogue scale (“How lucky did you feel?” with 1 indicating *extremely unlucky* and 9 indicating *extremely lucky*). No time constraints were imposed on bet selection or luck ratings. During a variable intertrial interval (8–12 s), only a fixation cross was displayed, to allow for recovery of physiological signals.

Three different wheel types were presented to manipulate the outcome, in a pseudorandomized sequence. The key wheel of interest contained both win (+10×) and loss segments (−10×), thus offering a neutral expected value. The other two wheel types offered only a win or loss segment (and therefore the possibility of delivering only near-wins and full-misses, or near-losses and full-misses, respectively), generating a positive and negative expected value on those wheels. These were included in order that participants should vary their bet on a trial-by-trial basis (see the results in the online supporting information). The outcomes were fair, such that each segment was selected five times, with wins and losses on one in eight trials (12.5%), and near-wins, near-losses, and full-misses each occurring on two in eight trials (25%). Near-wins were zero outcomes in the segment either side of the win. Similarly, near-losses were zero outcomes in the segment either side of the loss. Both near events were compared against a baseline of “full-misses,” where the highlighted null segment was not adjacent to either the win or loss segment. An equal number of near-wins and near-losses were delivered on either side of the win and loss, respectively.

Following the gambling task, participants viewed screenshots of near-wins that stopped before and after the winning segment, and near-losses that stopped before and after the losing segment. For each screenshot, they were asked to list their first thought about how the outcome could have been different.

### Facial EMG Measurement

Facial EMG data were collected via a BIOPAC (Santa Barbara, CA) MP36R, recording at 1,000 samples per second. The BIOPAC was connected to a stimulus delivery computer and a second administrator computer running Acqknowledge v4.1. Events occurring on the stimulus delivery computer (including the outcomes on the task) were synchronized to the facial EMG recording using digital channels. Facial EMG recordings were collected through 4-mm shielded chloride electrodes attached to the skin over the left eye (i.e., corrugator) and left cheek (i.e., zygomaticus muscles) via 4-mm adhesive disks, according to the standard procedures established previously (Fridlund & Cacioppo, 1986). Following attachment of fEMG electrodes, 5 min of resting state data were acquired, before the instructions for the wheel of fortune task were read to the participant.

### Data Processing and Analysis

Data were screened prior to analysis and resampled at 100 Hz. The raw fEMG data, recorded at 5–500 Hz, were extracted using an inhouse script programmed in R Studio (R Development Core Team, 2008). The data were filtered through a 30 Hz high-pass filter to remove low frequency noise and artifacts recorded during the task. The filtered data were then rectified, converting negative values into positive values. Mean values were extracted for a baseline period in the final 2 s of the spin, and for 4 s following the wheel stopping (the outcome). Percentage change from baselines was calculated, in order to compare activity at the two muscle locations.

For the behavioral data, we used R and *nlme* (Pinheiro, Bates, DebRoy, Sarkar, & R Development Core Team, 2013) to perform a linear mixed effects analysis on the two main dependent variables: (1) luck ratings, (2) the change in the bet amount from the current trial, *n,* to the next trial, *n* + 1. We use linear mixed effects (LME) modeling via restricted maximum likelihood for all repeated measures analyses to reduce information loss when evaluating large, unbalanced data sets after signal standardization (Carr et al., 2013; Judd, Westfall, & Kenny, 2012). As a random effect, we had an intercept representing participant number. For each dependent variable, we ran three separate models. In the first model, we assessed the impact of the objective outcomes as a fixed effect, with three levels to compare wins, losses, and null outcomes. In the second model, we compared the three types of null outcomes (i.e., near-wins, near-losses, and full-misses). In the third model, we considered near-miss type (i.e., near-win, near-loss) as well as its position (i.e., whether the segment stopped just before or passing through the win/loss segment), treating both factors as fixed effects (with interaction terms). Visual inspection of residual plots did not reveal any obvious deviations from homoskedasticity or normality. For all the models on luck ratings, the bet amount at the start of the current trial (i.e., before the outcome was delivered) was entered as a fixed factor of no interest. To assess the validity of the mixed effects analyses, we performed likelihood ratio tests comparing the models with fixed effects to the null models with only the random effects. We rejected results in which the model including fixed effects did not differ significantly from the null model.

For the facial EMG data, we averaged the raw data under each experimental condition. The LME model was used with participant number entered as a random effect factor, using the equivalent three sets of models to the behavioral data.

## Results

### Manipulation Check

We coded the counterfactual statements given by the participants as +1 for an upward counterfactual (e.g., “I could have won a lot of money”), 0 for no counterfactual (“I don't mind”), −1 for a downward counterfactual (e.g., “I could have lost 10 times the amount I bet”; based upon Wohl & Enzle, 2003). These were coded by two independent judges who were blind to the purpose of the study, and interrater agreement rate was 100%. A 2 (Type: near-wins vs. near-losses) × 2 (Position: before vs. after segment) repeated measures analysis of variance (ANOVA) showed a significant main effect of near-miss type, *F*(1,44) = 241.76, *p* < .001, 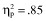. Near-wins elicited upward counterfactuals, *M* = 0.70, *SD* = 0.42, whereas near-losses elicited downward counterfactuals, *M* = −0.82, *SD* = 0.32. Neither the main effect of near-miss position nor the interaction term were statistically reliable, both *F*(1,44) < 1.

### Luck Ratings

The first model assessed the impact of the different objective outcomes (three levels: win vs. loss vs. null) on the luck ratings (see Table 1). There was a significant main effect of outcome type, χ^2^(2) = 146.12, *p* < .001, with participants feeling luckier following wins compared to null outcomes, *b* = 1.93, *t*(88) = 7.61, *p* < .001, and following null outcomes compared to losses, *b* = −2.31, *t*(88) = −9.12, *p* < .001.

**Table 1:** Behavioral Responses to the Objective Gains and Losses on the Wheel of Fortune Task [Mean (SD)]

	Win	Loss	Null
Luck rating	6.88 (1.27)	2.62 (1.68)	4.95 (0.63)
Betting amount change	−4.84 (12.06)	−0.18 (9.23)	1.20 (2.41)

The second model compared the three types of null outcomes (i.e., near-wins vs. near-losses vs. full-misses; see Table 2). There was a significant main effect of outcome type, χ^2^(2) = 30.13, *p* < .001. Near-losses significantly increased luck ratings relative to full-misses, *b* = 0.29, *t*(88) = 2.91, *p* < .01. Conversely, luck ratings were lower following near-wins compared to full-misses, *b* = −0.31, *t*(88) = −3.06, *p* < .01.

**Table 2:** Behavioral Responses to the Null Outcomes, Comparing the Near Events Against Full-Misses [Mean (SD)]

	Near-wins	Near-losses	Full-misses
Luck rating	4.65 (0.81)	5.24 (0.72)	4.95 (0.69)
Betting amount change	1.89 (5.89)	0.78 (5.25)	0.93 (3.91)

The near-loss effect was further moderated by the trait level of beliefs in luck, on the BIGL scale. The BIGL score was positively correlated with the increase in luck ratings following a near-loss compared to a full-miss, *r* = 0.37, *p* = .01. The BIGL score did not predict the luck ratings following full-misses, *r* = −.22, *p* > .1, or the decrease after near-wins compared to full-misses, *r* = −.20, *p* > .1. GRCS scores were moderately correlated with the BIGL scores (Darke & Freedman, 1997b), *r* = .39, *p* < .01, and with the trial-by-trial luck ratings averaged across all the experimental conditions, *r* = .34, *p* < .05, but the GRCS scores did not predict the luck ratings in any single experimental condition (e.g., near-wins or near-losses, *p*s > .1).

The third model decomposed the four types of near-misses by near-miss type (near-wins vs. near-losses) and near-miss position (before vs. after). The main effect of near-miss type was already established in the second model, and, similar to the manipulation check for counterfactual thinking, neither the main effect of near-miss position nor the interaction term reached significance, both χ^2^(1) < 1.

### Bet Amount Change

In the first model looking at the objective outcomes (see Table 1), there was a significant main effect of outcome type, χ^2^(2) = 11.23, *p* < .01, with participants reducing their bet following wins, compared to both null outcomes, *b* = −4.67, *t* = −2.49, *p* = .01, and losses, *b* = −6.04, *t* = −3.23, *p* < .01.

In the second model (see Table 2), there were no differences in betting behavior following the different types of null outcome, χ^2^(2) = 1.28, *p* > .1. Given this result, the third model decomposing the null events by position was not run on betting behavior.

### Facial EMG

#### Zygomaticus reactivity

**Figure 2:**
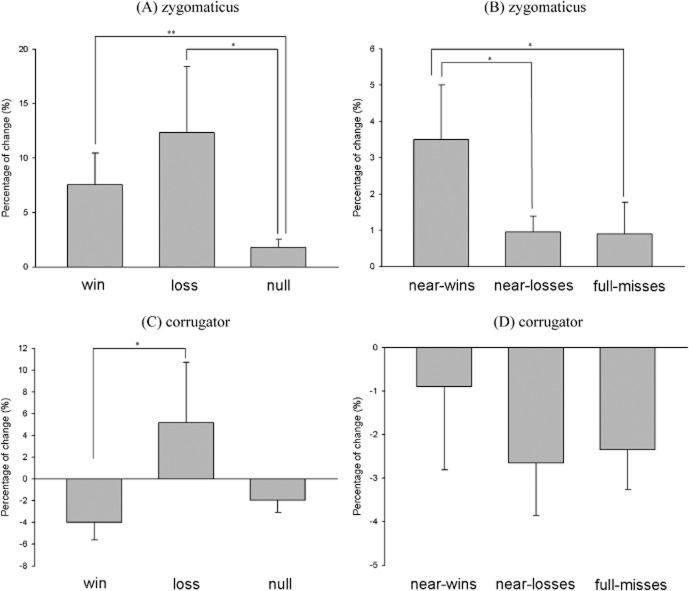
A: Zygomaticus reactivity to the objective gains and losses. B: Zygomaticus reactivity to the null outcomes, comparing the near events against full-misses C: Corrugator reactivity to the objective gains and losses. D: Corrugator reactivity to the null outcomes, comparing the near events against full-misses. Error bars represent standard errors of the mean.

The first model assessing the objective outcomes (see Figure 2A) yielded a marginally significant main effect, χ^2^(2) = 4.45, *p* = .1, which was driven by losses (*M* = 12.34%, *SD* = 40.65%) significantly increasing zygomaticus reactivity compared to null outcomes (*M* = 1.79%, *SD* = 4.99%), *b* = 10.55%, *t*(88) = 2.11, *p* < .05. An a priori test comparing zygomaticus activity following wins (*M* = 7.55%, *SD* = 19.53%) and null outcomes confirmed a significant response to wins, χ^2^(1) = 5.82, *p* = .01. There was no difference between wins versus losses, *b* = −4.79%, *t*(44) = −0.81, *p* > .1.

The second model tested for differences between three types of null outcomes (see Figure 2B). There was a significant main effect of outcome type, χ^2^(2) = 5.70, *p* = .05, with near-wins (*M* = 3.50%, *SD* = 2.87%) eliciting higher zygomaticus activity than both near-losses (*M* = 0.96%, *SD* = 10.06%), *b* = 2.54%, *t*(88) = 2.05, *p* < .05, and full-misses (*M* = 0.90%, *SD* = 5.83%), *b* = 2.60%, *t*(88) = 2.10, *p* < .05.

The third model confirmed the main effect of near-miss type in model 2, but neither the main effect of near-miss position, χ^2^(1) = 0.21, *p* > .1, nor the interaction term, χ^2^(1) = 0.007, *p* > .1, was significant.

#### Corrugator reactivity

In the first model looking at the objective outcomes (see Figure 2C), we found a marginally significant main effect of outcome type, χ^2^(2) = 4.50, *p* = .10, driven by losses (*M* = 5.16%, *SD* = 36.37%) significantly increasing corrugator reactivity relative to wins (*M* = −3.98%, *SD* = 10.88%), *b* = 9.14%, *t*(88) = 2.02, *p* < .05, consistent with our a priori prediction.

In the second model (see Figure 2D), there was no statistically reliable main effect of the null event type (near-wins: *M* = −0.90%, *SD* = 12.82%; near-losses: *M* = −2.65%, *SD* = 8.07%; full-misses: *M* = −2.35%, *SD* = 6.13%), χ^2^(2) = 1.67, *p* > .1, and therefore the third model further decomposing near events by position was not performed.

Thus, both objective wins and losses increased zygomaticus activity, whereas corrugator activity was selectively sensitive to losses. Near-wins increased zygomaticus but not corrugator activity, thereby showing a similar pattern to the actual wins. As a direct test of the similar profile, we ran a supplementary model to compare near-wins, objective wins, and objective losses. For the zygomaticus, near-wins and wins did not differ from each other, *b* = 4.79%, *t*(88) = 1.03, *p* > .1, and losses elicited stronger responses compared to near-wins, *b* = 8.84%, *t*(88) = 2.05, *p* < .05. For the corrugator, there was no difference between wins and near-wins, *b* = 3.08%, *t*(88) = 0.83, *p* > .1, and losses elicited greater responses compared to both objective wins, *b* = 9.14%, *t*(88) = 2.19, *p* < .05, and at trend, near-wins, *b* = 6.06%, *t*(88) = 1.63, *p* = .1.

## Discussion

By using a wheel of fortune task, the present study investigated the effects of near-wins and near-losses on self-perceived luck, betting behavior, and facial muscle reactivity. Subjectively, participants reported greater feelings of luckiness following near-losses, relative to full-misses, and this effect was further correlated with the trait beliefs in luck using Darke and Freedman's BIGL scale. Near-wins exerted the opposite effect on luck ratings, decreasing self-reported luck. While this effect did not scale with BIGL score, it is notable that the BIGL selectively assays positive aspects of luck. Betting behavior was primarily sensitive to the objective outcomes on the task, with participants reducing the amount of the bet following wins. Near-wins and near-losses had no significant influence on this adjustment in betting behavior in the next round of the game.

Our facial EMG data provide an important proof-of-principle demonstration for the differential sensitivities of zygomaticus and corrugator. Zygomaticus activity increased after objective wins and losses, whereas corrugator activity selectively increased following objective losses. Near-win outcomes significantly increased zygomaticus activity, relative to both full-misses and near-losses, but did not affect corrugator response, thus showing a similar response pattern as actual wins. This interpretation was confirmed in a model that directly compared objective wins and losses against near-wins.

Previous facial EMG research indicates that zygomaticus activity is a sensitive marker of appetitive processing (Cacioppo et al., 1986; Lang et al., 1993) and that corrugator activity is sensitive to aversive processing (Lang et al., 1993; Larsen et al., 2003). Thus, a simple interpretation of the zygomaticus reactivity to near-wins is that this reflects the appetitive nature of nearly winning, consistent with prior studies taking motivational ratings (Clark et al., 2009) and measurements of play duration (Côté et al., 2003; Kassinove & Schare, 2001). However, this interpretation is complicated by our observation that zygomaticus activity also increased following losses. We note that some past work has described zygomaticus activity to affective images varying as a quadratic function with emotional valence, such that both intensely positive and negative stimuli can enhance zygomaticus activity (Lang et al., 1993; Larsen et al., 2003). Critically, negative emotional stimuli also elicit a reliable effect on the corrugator, which was seen for objective losses in the present study but not for near-wins or objective wins. Thus, the combined pattern across the two sites for near-wins in the present study is most consistent with an appetitive signal—that near-wins engender some of the appetitive processing associated with actual wins.

The finding that near-losses increased luck ratings relative to full-misses corroborates Wohl and Enzle's (2003) finding that near-losses heightened perception of personal luck. Using a one-trial task with a subsequent risk decision on a different gamble, Wohl and Enzle (2003) observed no significant effect of near-wins, whereas in the current multishot task the effects of near-wins on luck perceptions mirrored the effects of near-losses (i.e., lower luck ratings after near-wins). It has been shown that emotional responses following counterfactual thinking are affected by the direction of the counterfactuals (Markman & McMullen, 2003; Roese, 1994). While comparing reality to a more desirable alternative can elicit more negative emotions (i.e., upward counterfactuals), comparing reality to a less desirable alternative can elicit more positive emotions (i.e., downward counterfactuals). Moreover, the counterfactual thinking is more likely to be mentally constructed when the reality and its alternative are in short distance (the “simulation heuristic”). This proximity could be of spatial (Johnson, 1986; Miller & McFarland, 1986), temporal (Macrae, Milne, & Griffiths, 1993), or numeric form (Medvec, Madey, & Gilovich, 1995; Medvec & Savitsky, 1997). Therefore, in the present study, being close to desirable but unrealized wins could encourage people to compare the reality to what could have been better (i.e., upward counterfactual comparison), as confirmed by the manipulation check, and this would make individuals feel unlucky. As suggested by previous near-miss research (Clark et al., 2009, 2013), this would elicit negative emotions such as frustration and disappointment. On the other hand, being close to an undesirable but averted loss could encourage people to compare the reality to what could have been worse (i.e., downward counterfactual comparison), as confirmed by the manipulation check, and this would make people feel luckier and give rise to positive emotions such as relief.

In betting behavior, we primarily observed an adjustment of betting following the objective outcomes: betting was reduced on trials following major wins compared to the other objective outcome types. This effect is consistent with a broad definition of the gambler's fallacy that people do not expect runs of consecutive identical outcomes (in this case, wins) in a random task (Ayton & Fischer, 2004). Sundali and Croson (2006) refer to this as the “stock of luck” belief, that good luck is exhaustible, and therefore people may strategically reduce their bet following wins given a perceived reduction in the probability of winning on the next trial. In the present study, this effect was asymmetrical, with no corresponding increase in betting observed following losses.

Despite the marked adjustment of betting following wins, we did not observe any reliable change in betting following near-wins or near-losses. As such, our results do not replicate Wohl and Enzle (2003), who reported increased wagering in a group who experienced near-losses, compared to a group who experienced near-wins. There are several pertinent methodological differences that may account for the discrepancy: Wohl and Enzle's (2003) study involved single rounds of two different gambling tasks, whereas we employed a multishot version of a single gambling task, in which learning and habituation may reduce carryover effects of near events on subsequent risk taking. Another study using scenarios also found that narrowly avoiding a disaster (i.e., a near-loss) reduced people's risk perceptions, and increased subsequent risky choices (Dillon & Tinsley, 2008).

In the present study, we did not find any position effects of near events either side of the win/loss segment, either on subjective luck ratings or on facial muscle reactivity. Using a slot machine simulation, Clark et al. (2013) previously observed that the motivational effect of near-wins was restricted to those events that stopped before the payline, whereas near-wins after the payline were primarily aversive (but see Wohl & Enzle, 2003 Experiment 2). These differences are compatible with theories of counterfactual thinking (Markman & McMullen, 2003), but may be somewhat fragile and depend upon the precise temporal dynamics of the anticipatory period, or differential sensitivities of the luck rating as the dependent variable here. One limitation of the present study is that the luck rating and bet adjustment variables do not map directly to the ratings of pleasantness and motivation used in some past work. Our results also failed to corroborate a previous study by Bediou et al. (2011) in which zygomaticus activity was greater following gains relative to losses. In our study, financial losses also elicited zygomaticus activity. We note that the Bediou et al. (2011) experiment used a social competition task, and also did not include null financial outcomes as a baseline. We would encourage further work recording facial EMG during tasks of economic decision making.

To our knowledge, this is the first study using facial EMG to investigate near-miss effects in a laboratory setting. Zygomaticus was sensitive to objective wins and losses, whereas corrugator was only sensitive to objective losses. Near-wins were perceived as unlucky, but heightened zygomaticus activity, showing a similar facial muscle response pattern as actual wins. This is consistent with previous literature showing the bivalent emotional nature of near-wins. This supports the utility of facial EMG as a marker of emotional reactivity in gambling and decision-making research. Near-wins and near-losses elicited downward and upward counterfactuals, respectively, and drove luck perceptions in opposing directions, which emphasizes the role of counterfactual thinking in the near-miss effect.

## Footnotes

1 This is a slightly unorthodox form of “bet” where, on the null outcome, the wager was effectively returned to the subject. That is to say, the bet only served as a multiplier on actual win or loss outcomes.

## Supporting Information

**Appendix S1:** Results on Wheel A and Wheel B **Figure S1:** Wheel A **Figure S2:** Wheel B
