# Supplementary material for: Near-wins and near-losses in gambling: A behavioral and facial EMG study
Source: Psychophysiology. 2014 Sep 19;52(3):359–66. doi: 10.1111/psyp.12336 (PMC4510820; doi:10.1111/psyp.12336)

Besides the critical wheel type that is presented in the main report, the wheel of fortune presented two further wheel types, in order to encourage the participants to vary their betting amount on a trial-by-trial basis. One wheel type was of negative expected value (EV), and the other one of positive EV (see Fig. S1). These two wheels were mixed with the critical wheel (EV = 0). We present the behavioral and fEMG results of these two wheels here.

**Wheel A**

On wheel A (see Fig. S1), there were six small win segments on which participants could win the same size of the bet if one of them were highlighted at the end of wheel deceleration, and two big loss segments on which they could lose 10 times of the bet. Forty trials terminated an equal number of times in each segment, such that there were 25% big losses, 25% near-losses (the two small win segments that were adjacent to the loss segments) and 50% small wins (the four small win segments that were in two or more position from the loss segment) on this wheel.

*Luck ratings*

There was a significant main effect of outcome type on luck rating, *χ^2^*(2) = 123.16, *p* < 0.001, with both small wins (*M* = 5.75, *SD* = 0.77) and near-losses (*M* = 5.89, *SD* = 0.87) increasing luck feelings compared to big losses (*M* = 2.86, *SD* = 1.64), *b* = 2.88, *t*(88) = 12.41, *p* < 0.001, and *b* = 3.02, *t*(88) = 12.84, *p* < 0.001, respectively. There was no difference between small wins and near-losses, *b* = 0.14, *t*(88) = 0.60, *p* > 0.1.

*Bet amount change*

There was a marginally significant main effect of outcome type, *χ^2^*(2) = 5.23, *p* = 0.07, with losses (*M* = 2.88, *SD* = 9.27) increasing bet amount change compared to both small wins (*M* = –0.12, *SD* = 5.55) and near-losses (*M* = –0.11, *SD* = 6.18), *b* = 3.00, *t*(88) = 1.98, *p* = 0.05, and *b* = 2.99, *t*(88) = 1.97, *p* = 0.05, respectively. No difference was found between small win and near-losses, *b* = 0.01, *t*(88) = 0.01, *p* > 0.1.

*Zygomaticus reactivity*

There was a significant main effect of outcome type on zygomaticus reactivity, *χ^2^*(2) = 7.60, *p* < 0.05. Big losses (*M* = 7.51%, *SD* = 16.04%) increased zygomaticus reactivity compared to both small wins (*M* = 2.32%, *SD* = 5.87%) and near-losses (*M* = 3.05%, *SD* = 8.14%), *b* = 5.19%, *t*(88) = 2.57, *p* = 0.01, and *b* = 4.45%, *t*(88) = 2.21, *p* < 0.05, respectively. No difference was found between small wins and near-losses, *b* = 0.73%, *t*(88) = 0.36, *p* > 0.1.

*Corrugator reactivity*

There was no significant main effect of outcome type on corrugator reactivity, *χ^2^*(2) = 1.20, *p* > 0.1.

*Summary*

On wheel A, near-losses did not differ from small wins (baseline) on luck ratings, suggesting that the manipulation of near-losses did not work very well on this wheel. Wins reduced bet amount change compared to losses, consistent with the “stock of luck belief” found on the critical wheel. Big losses increased zygomaticus reactivity relative to wins, which is similar to the pattern observed on the critical wheel.

**Wheel B**

On wheel B (see Fig. S2), there were six small loss segments on which participants could lose the same size of the bet if one of them were highlighted at the end of wheel deceleration, and two big win segments on which they could win 10 times of the bet. Forty trials terminated an equal number of times in each segment such that there were 25% big wins, 25% near-wins (the two small loss segments that were adjacent to the win segments) and 50% small losses (the four small loss segments that were in two or more position from the win segments) on this wheel.

*Luck ratings*

There was a significant main effect of outcome type, *χ^2^*(2) = 133.01, *p* < 0.001. Both small losses (*M* = 4.03, *SD* = 0.91) and near-wins (*M* = 3.91, *SD* = 0.91) significant decreased luck ratings compared to the big wins (*M* = 6.85, *SD* = 1.26), *b* = –2.82, *t*(88) = –12.84, *p* < 0.001, and *b* = –2.94, *t*(88) = –13.23, *p* < 0.001, respectively. There was no difference between small losses and near-wins, *b* = –0.13, *t*(88) = –0.58, *p* > 0.1.

*Bet amount change*

There was a significant main effect of outcome type on bet amount change, *χ^2^*(2) = 17.05, *p* < 0.001, with big wins (*M* = –4.33, *SD* = 9.28) reducing bet amount change relative to both small losses (*M* = 0.63, *SD* = 4.71) and near-wins (*M* = 1.24, *SD* = 5.84), *b* = –4.97, *t*(88) = 3.42, *p* < 0.001, and *b* = –5.58, *t*(88) = –3.84, *p* < 0.001, respectively. However, there was no difference between near-win and small loss, *b* = 0.61, *t*(88) = 0.42, *p* > 0.1.

*Zygomaticus reactivity*

There was no main effect of outcome type on zygomaticus reactivity, *χ^2^*(2) = 0.49, *p* > 0.1.

*Corrugator reactivity*

There was a significant main effect of outcome type on corrugator reactivity, *χ^2^*(2) = 10.98, *p* < 0.01, with wins (*M* = –6.54%, *SD* = 10.28%) deactivating corrugator reactivity relative to small losses (*M* = –2.27%, *SD* = 7.99%) and near-wins (*M* = –2.24%, *SD* = 6.83%), *b* = –4.27%, *t*(88) = –2.92, *p* < 0.01, and *b* = –4.30%, *t*(88) = –2.94, *p* < 0.01, respectively.

*Summary*

On wheel B, near-wins did not differ from small losses (baseline) on luck ratings, suggesting that the manipulation of near-wins did not work very well on this wheel. Wins reduced bet amount change compared to losses, consistent with the “stock of luck belief” seen on the critical wheel. Wins deactivated corrugator reactivity relative to losses, which is similar to the pattern observed on the critical wheel.

**Figure S1**. Wheel A


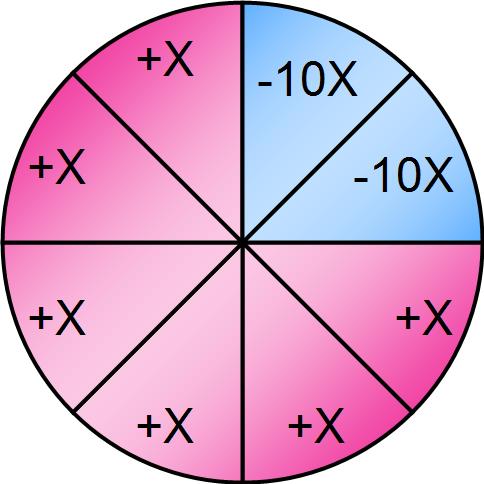


**Figure S2**. Wheel B


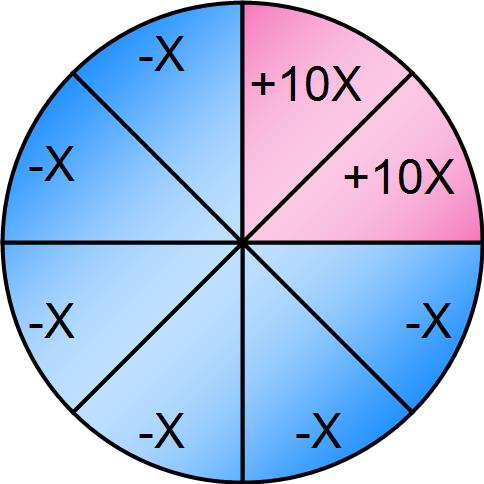

Supplement: Supplementary file 1 [file psyp0052-0359-sd1.docx]
